# Effect of gamma irradiation on proliferation and growth of friable embryogenic callus and *in vitro* nodal cuttings of ugandan cassava genotypes

**DOI:** 10.3389/fpls.2024.1414128

**Published:** 2024-09-16

**Authors:** Hellen B. Apio, Wilfred Elegba, Wonder Nunekpeku, Solomon Ayeboafo Otu, Julius Karubanga Baguma, Titus Alicai, Kenneth Ellis Danso, Isaac Kofi Bimpong, Emmanuel Ogwok

**Affiliations:** ^1^ Tissue culture and Transformation Laboratory, National Crops Resources Research Institute (NaCRRI), Kampala, Uganda; ^2^ Biotechnology and Nuclear Agriculture Research Institute (BNARI), Ghana Atomic Energy Commission (GAEC), Accra, Ghana; ^3^ School of Nuclear and Allied Sciences, University of Ghana, Accra, Ghana; ^4^ Plant Breeding and Genetics Section, Joint Food and Agricultural Organisation (FAO)/International Atomic Energy Agency (IAEA) Centre of Nuclear Techniques in Food and Agriculture, Vienna, Austria; ^5^ Department of Science and Vocational Education, Faculty of Science, Lira University, Lira, Uganda

**Keywords:** cassava genotypes, gamma radiation, friable embryogenic callus, *in vitro* nodal cuttings, mutation induction

## Abstract

Cassava (*Manihot esculenta Crantz*) production and productivity in Africa is affected by two viral diseases; cassava mosaic disease (CMD) and cassava brown streak disease (CBSD). Induced mutagenesis of totipotent/embryogenic tissues or *in vitro* plant material can lead to the generation of CMD and/or CBSD tolerant mutants. To massively produce non-chimeric plants timely and with less labor, totipotent cells or tissues are a pre-requisite. This study aimed to determine the effect of gamma radiation on the proliferation and growth of friable embryogenic callus (FEC) and *in vitro* nodal cuttings respectively. To obtain FEC, 2-6 mm sized leaf lobes of nine cassava genotypes were plated on Murashige and Skoog (MS) basal media supplemented with varying levels (37, 50, 70, 100) μM of picloram for production of organized embryogenic structures (OES). The OES of five cassava genotypes (Alado, CV-60444, NASE 3, NASE 13 and TME 204) were crushed and plated in Gresshoff and Doy (GD) basal media in combination with the amino acid tyrosine in varying concentrations for FEC production. FEC from five cassava genotypes and *in vitro* nodal cuttings of nine genotypes were irradiated using five different gamma doses (0, 5, 10, 15, 20 and 25 Gy) at a dose rate of 81Gy/hr. The lethal dose (LD)50 was determined using the number of roots produced and flow cytometry was done to determine the ploidy status of plants. The highest production of OES was noted in Alado across varying picloram concentrations, while TME 204 obtained the highest amount of FEC. The irradiated FEC gradually died and by 28 days post irradiation, FEC from all five cassava genotypes were lost. Conversely, the irradiated in vitro nodal cuttings survived and some produced roots, while others produced callus. The LD50 based on number of roots varied from genotype to genotype, but plants remained diploid post-irradiation. Accordingly, the effect of gamma irradiation on Ugandan cassava genotypes (UCGs) was genotype-dependent. This information is foundational for the use of *in vitro* tissues as target material for cassava mutation breeding.

## Introduction

Cassava (*Manihot esculenta* Crantz) is an important dietary carbohydrate source for approximately 800 million people in the tropics. In Uganda, it serves as food security and an income generation crop for both the rural and urban populations. The crop is highly heterozygous, with low pollen fertility, low fruit set and a long breeding cycle (Ceballos et al., 2004; Bull et al., 2017; Ceballos et al., 2020). Cassava production is also highly affected by two viral diseases: cassava brown streak disease (CBSD) and cassava mosaic disease (CMD). CBSD is caused by two single-stranded RNA viruses, Cassava brown streak virus (CBSV) and Ugandan cassava brown streak virus (UCBSV) of the family *Potyviridae* and genus *Ipomovirus* (Mbanzibwa et al., 2009; Monger et al., 2010; Winter et al., 2010). CMD is caused by cassava mosaic geminiviruses (CMG) of the family *Geminiviridae* and genus *Begomovirus* (Legg and Thresh, 2000; Tembo et al., 2024), are single-stranded DNA bipartite CMGs. The two diseases are transmitted by the whitefly (*Bemisia tabaci*) (Maruthi et al., 2005; Mware et al., 2009), and can result in 100% yield losses (Alicai et al., 2007). The diseases are also spread through the use of infected planting materials (Patil et al., 2015; Robson et al., 2024).

The symptoms associated with CBSD in cassava are; leaf chlorosis, brown streaks on stems, and brown necrosis on storage roots, resulting in a decrease in root weight and starch content, ultimately, root yield and root quality (Hillocks and Jennings, 2003; Sheat et al., 2019). In the case of CMD, the storage root yield or starch content is reduced due to the chlorotic and mottled nature of the leaves affecting photosynthesis in the crop (Elegba et al., 2013; Mukiibi et al., 2019). Conventional breeding approaches to develop resistance to cassava brown streak disease (CBSD) have been limited due to lack of genetic resistance in cultivated cassava genotypes. Genetic engineering (GE) is being used to complement conventional breeding (CB) due to the crop’s long breeding cycles and high heterozygosity. Strategies such as RNA interference (RNAi) or gene silencing have been used to develop transgenic plants conferring resistance to cassava brown streak disease and have been tested and proven to provide resistance to the cassava brown streak viruses (Wagaba et al., 2016). However, due to the misconstrued conceptions about GE in the public domain, many find it challenging to embrace the technology (Zawedde et al., 2018).

Mutagenesis has been used as an alternative strategy to introduce desirable traits into several crop species including cassava resulting in the release of over 3500 mutant varieties (Forster and Shu, 2012; Danso and Elegba, 2017; Kharkwal, 2023).The formation of mutations in plants can occur as a result of exposure to DNA-damaging (genotoxic) agents, or it can occur spontaneously in cells. Chemical and physical mutagens have been found to cause sudden heritable changes in the genetic information of plants (Ulukapi and Gul Nasircilar, 2018). In particular, gamma irradiation of cassava stem cuttings led to the release of two mutant cassava varieties named “Tekbankye” in Ghana in 1997, and “Fuxuan” in China in 2005 respectively (Forster and Shu, 2012; Danso and Elegba, 2017) indicating that gamma radiation penetrates bulky cassava stem cuttings (Asare and Safo-Kantanka, 1995; Danso and Elegba, 2017). Gamma-irradiated stem cuttings of Jame-jame and Adire-4, resulted in changes in the tuber shape, size and color (Khumaida et al., 2015). Induced mutagenesis has also led to the modification of several agronomic traits such as lodging resistance, early maturity, disease resistance and product quality (e.g., protein and lysine content) in several crops (Nencheva, 2010; Oldach, 2011; Leitao, 2012; Oladosu et al., 2016) leading to the release of crop varieties with improved agronomic traits and tolerance to ecological stresses (Mullins et al., 2021).

It is highly likely that irradiation of cassava stem cuttings results in the production of chimeric mutant plants due to the use of multi-cellular plant parts, mainly cassava stakes as start material for mutation induction (Asare and Safo-Kantanka, 1995; Khumaida et al., 2015). Chimerism refers to the accumulation of different mutation events in different cells of a plant propagule (Frank and Chitwood, 2016; Datta et al., 2018) and requires several cycles of propagation to produce non-chimeric and stable mutant plants. In a bid to massively produce non-chimeric plants timely and with less labor, totipotent cells or tissues are a pre-requisite. Production of totipotent cells or tissues in cassava has been made possible using tissue culture techniques referred to as somatic embryogenesis (SE) and micropropagation respectively (George et al., 2008; Apio et al., 2015; Elegba et al., 2021). Somatic embryogenesis involves the generation of organized embryogenic structures (OES) and friable embryogenic callus (FEC), which can dedifferentiate into totipotent embryonic tissues giving rise to an embryo and a fully grown plant under appropriate conditions (Taylor et al., 2004; Apio et al., 2015; Elegba et al., 2021).

Micropropagation facilitates the rapid multiplication of nodal cuttings *in vitro*, under appropriate conditions, bulking up planting materials all year round (George et al., 2008; Apio et al., 2015). Irradiation of OES, FEC or *in vitro* nodal cuttings can induce mutations in cassava facilitating the development of massive populations of mutant cassava varieties with desirable attributes (López et al., 2017) including resistance to CMD and/or CBSD in Africa. Cassava is a diploid species (2n = 36) (Soto et al., 2015). Gamma irradiations have been documented to make changes in the genome of the cells or tissues ranging from single-stranded breaks (SSBs), double-stranded breaks (DSBs) insertion, deletions or transversions (Du et al., 2022). Determination of changes in the genome and ploidy can be conducted using flow cytometry (FC), a high-throughput technique that provides accurate results at low costs (Garavello et al., 2019). FC utilizes the nucleic acid-specific fluorochrome propidium iodide for the analysis of DNA content and ploidy levels because it’s a rapid, simple, and reproducible approach (Escobedo-Gracia-Medrano et al., 2018). The relative DNA content of each stained nucleus is quantified using lasers that emit the fluorescence.

In this study, *in vitro* nodal cuttings and FECs were exposed to different doses of gamma radiation, to develop mutant cassava varieties with combined resistance to CMD and CBSD in selected Ugandan cassava genotypes (UCGs). To achieve this, we specifically determined the effect of auxins and amino acids on the production of organized embryogenic structures (OES) and friable embryogenic callus (FEC) and investigated the effect of gamma radiation on FEC and *in vitro* nodal cuttings of UCGs. The effect of gamma irradiation on the growth and survival of *in vitro* nodal cuttings and FEC explants was characterized using the flow cytometry technique.

## Materials and methods

### Production of organized embryogenic structures

A total of nine genotypes namely; NASE 3, NASE 12, NASE 13, NASE 19, Alado, TME 204, CV-60444, NAROCASS 1 and NAROCASS 2 were used for this experiment. The genotypes were selected for their resistance to cassava mosaic disease (CMD) (NASE 3, NASE 12, NASE 13, TME 204), tolerance to cassava brown streak disease (CBSD) (NASE 19, NAROCASS 1, NAROCASS 2) and susceptibility to both diseases (Alado, CV-60444). The experiment was conducted in the tissue culture laboratory of the National Crops Resources Research Institute (NaCRRI), Uganda using a completely randomized design (CRD). A total of 27 Petri dishes, each containing 9 explants (leaf lobes) per Petri dish, amounting to a total of 243 leaf lobes were excised and placed on Murashige and Skoog (MS) media (Murashige and Skoog, 1962) with varying concentrations (37, 50, 70 or 100 µM) of picloram for the production of OES (Taylor et al., 1996; Nyaboga et al., 2015). The placed leaf lobes of each genotype were monitored on the MS medium for 28 days to ascertain the ability of each genotype to produce OES with varying picloram concentrations. Data on frequency and surface area coverage of OES were collected. The OES produced from each genotype was collected and placed on sterile stainless-steel mesh. The mesh was then placed over a 90 x 15 cm Petri dish containing Gresshoff and Doy (GD) basal media (Gresshoff and Doy, 1974) supplemented with 20 g of sucrose, 50 µM picloram and 3g of Gelzan to minimize moisture loss in the explants. The OES was crushed using a sterile spatula and collected in the aforementioned medium. The crushed OES was then plated on GD basal media supplemented with 50µm picloram and 10 mM stock of the amino acid, tyrosine at varying concentrations (250 or 500 µM) for induction of friable embryogenic callus (FEC) in the UCGs. FEC induction in the control genotype, CV-60444 was on GD medium without tyrosine (Apio et al., 2015). A total of 10 Petri dishes, containing 5 clusters of crushed OES per plate, amounting to a total of 50 clusters per genotype were established at 28 ± 2°C. Data on frequency (number of explants that produced OES), surface area coverage (the amount of area covered by the OES produced per explant using a scale of +-20, ++-40, +++-60, ++++-80, +++++-100) and frequency of FEC produced from each clump of OES in each genotype was taken.

### The effect of gamma radiation on proliferation and growth of FEC

The experiment was conducted at the Biotechnology and Nuclear Agriculture Research Institute (BNARI), Ghana Atomic Energy Commission, Ghana using a completely randomized design (CRD). Five cassava genotypes; Alado, CV-60444, NASE 3, NASE 13 and TME 204 which produced sufficient amounts of FEC were used in the irradiation experiments. The FECs generated at NaCRRI, Uganda were transferred to BNARI, Ghana. The production of FEC was genotype-dependent resulting in variation in the quantities of FEC obtained. Thus, we used different quantities (2.1 g to 12 g) to ascertain the effect of gamma radiation on the proliferation and growth of FEC. 8.0 g of FEC was used for Alado, 2.1 g for TME 204, 6.0 g for NASE 3, 10 g for NASE 13 and 12 g for CV-60444. The FEC for each genotype was replicated three times using equal amounts in three Petri dishes for each radiation dose to ensure repeatability and uniformity of the sample size. The FEC from each genotype was placed on a sterile mesh to ensure that the FEC was not in direct contact with the media. The FEC on the mesh was moved to Petri dishes (90 x 15 cm) containing MS medium with 20 g of sucrose, and 3 g of Gelzan and sealed with cling film to prevent moisture loss in FEC. The Petri dishes containing the FEC on mesh were subjected to gamma radiation at five doses (5, 10, 15, 20 or 25 Gy) and the control at a dose rate of 81 Gy/hr at the Gamma Irradiation Facility (GIF)) using a Cobalt-60(^60^Co) machine. Five (5) colonies were established per Petri dish per treatment and the exposure time for each radiation dose varied. For 5 Gy, the exposure time was (3 mins 42 secs), 10 Gy (7 mins 24 secs), 15 Gy (11 mins 6 secs), 20 Gy (14 mins 48 secs) and 25 Gy (18 mins 30 secs). The irradiated FEC was transferred immediately to fresh MS medium supplemented with 13 µM or 27 µM of naphthalene acetic acid (NAA) and a control (without NAA) to facilitate recovery and growth of the irradiated FEC (Taylor et al., 1996; Apio et al., 2015). Responses of the FEC to the different radiation doses in culture were assessed weekly for 4 weeks.

### Histological observation by cross-section and transmission electronic microscopy

The mutagenized callus and the control were collected and fixed in formalin-acetic acid alcohol (FAA) for 12 hours at room temperature: 25°C using an automatic tissue processor (LEICA TP 1020, Germany) and then embedded in paraffin wax using a moulder (MEDAX GmbH & CO., Germany). Sections of 15µm thickness were cut using a manual rotary microtome (LEICA RM 2235, Germany) and placed on ordinary slides to fix the samples. After drying overnight at 63°C in the oven (Esco Isotherm), the samples were deparaffinized in xylene (www.merck-chemicals) for 20 minutes. The sections were rehydrated in 100% alcohol for 1-2 minutes, then 95% alcohol for 1-2 minutes. The sections were then rinsed in both tap water and distilled water and stained with hematoxylin (LOBA CHEMIE PVT LTD) for 3 - 5 minutes. The sections were differentiated with 1% HCl in 70% alcohol for two dips and checked under the microscope. The slides were washed in running tap water for 15 minutes and stained in Eosin for up to 4 minutes. The stained slides were dehydrated and differentiated by dipping 6 times in 95% alcohol before transferring to 100% alcohol and dipped 6 times. The slides were cleared two times in xylene and mounted by adding 100% alcohol for 3 minutes (mounting media) (Protocol-Histopathology laboratory, Veterinary medicine, Makerere University). The slides were then viewed using the transmission electron microscope (Nikon CSI –Japan) (CDL Laboratories, Veterinary Medicine, Makerere University).

### The effect of gamma radiation on growth of *in vitro* nodal cuttings

The parent material used for the establishment of *in vitro* cultures was screened for the presence of cassava mosaic disease (CMD) and cassava brown streak disease (CBSD) (Apio et al., 2021). Disease-free, two-month-old *in vitro* nodal cuttings of eight cassava genotypes (Alado, NASE 3, NASE 14, NASE 13, NASE 12, NASE 19, NAROCASS 1 and NAROCASS 2) from NaCRRI in Uganda were used in the experiment conducted at BNARI, Ghana. A total of 10 explants were used for each treatment per genotype. Five (5) nodal cuttings (1.0 - 1.5 cm) were placed in a 90 x 15 cm petri dish containing MS medium, with 20 g of sucrose, and 3 g of Gelzan, sealed with cling film to minimize moisture loss in the explants. Two Petri dishes each containing five nodal cuttings were set up for each radiation dose and genotype. The prepared *in vitro* nodal cuttings were exposed to gamma radiation at five doses (5, 10, 15, 20, 25 Gy) at the Gamma Irradiation Facility (GIF) using a Cobalt-60 (^60^Co) machine. The dose rate was determined for each of the five doses by calculating the dose per unit time in microSieverts and the samples exposed to the gamma source for varied exposure times as described in the preceding section The experiments were set up in a completely randomized design. The irradiated cultures from each genotype were transferred to MS medium supplemented with 20 g of sucrose and 3.0 g of Gelzan to allow for regeneration. The irradiated *in vitro* nodal cuttings were monitored for survival, root formation and shoot increase. Data was taken at 4, 7-, 14-, 21- and 28-days post irradiation.

### Flow cytometry determination of changes in nuclear genome ploidy of regenerated gamma-irradiated *in vitro* nodal cuttings

One-month-old *in vitro* nodal cuttings of the eight genotypes (Alado, NASE 3, NASE 14, NASE 13, NASE 12, NASE 19, NAROCASS 1 and NAROCASS 2), that survived the different gamma irradiation doses (5,10,15, 20, 25 Gy) and the control plants were used in this experiment. Plant material (nodal cuttings 0.2 cm in length) from each survived plant per dose was excised and placed in a 60 cm Petri dish and sealed. Five hundred microliters (500 µl) of Nucleic Acid Extraction buffer were added to each sample, chopped using a razor blade for one minute and incubated for 90 seconds. The samples were filtered using a 50 µm celltrics filter into a sample tube, and 2 ml of the staining solution (CyStain PI Absolute P) was added and incubated for 60 - 120 minutes protected from light at room temperature. The CyStain PI Absolute P is a fluorescent probe that binds to DNA. To have an overview of any introduced variation due to gamma irradiation in the nuclear genome ploidy levels of survived plants, all the survived plants per dose per genotype were pooled to make one sample. Six (6) samples per radiation dose for each genotype were analyzed using the Sysmex Partec GmbH flow cytometer to ascertain the ploidy status of the plants. This experiment was conducted in a completely randomized design. The prepared cell suspension sample for each dose rate was attached to the flow cytometer, which sucked up the sample and mixed it with the sheath fluid resulting in the formation of a single cell line which was analyzed by passing through a laser beam. The light from the single-cell line was scattered forward and sideways which was detected by the detector. The detector converted the scatter light into a voltage pulse which was directly proportional to the amount of forward scattered light and side scatter. The computer attached to the flow cytometer converts that data into a histogram that is proportional to the forward scatter and side scatter ascertaining the size and internal properties of the cell and enabling the determination of the ploidy status (Escobedo-Gracia-Medrano et al., 2018). The mean values produced by the Sysmex Partec flow cytometer and histograms generated were used to explain the changes observed.

### Determination of the lethal dose (LD_50_)

The LD_50_ (lethal dose), a dose that causes 50% lethality to the explants or the dose at which the irradiated *in vitro* plants recorded 50% growth performance (Oldach, 2011). The LD_50_ was estimated based on the number of roots produced by *in vitro* plantlets exposed to the different gamma radiation doses in comparison to the control (unirradiated *in vitro* nodal cuttings). More precisely, the LD_50_ value was graphically determined using the linear regression equation in Microsoft Excel (version 2010), by plotting the number of roots was plotted against the mutagen doses and the dose corresponding to a 50% reduction in the number of roots was read off the graph. The equation used: y = mx + c, where y= independent variable (usually corresponds to 50%), m = the slope or gradient, x = dependent variable, c = y intercept (value of y when x = 0).

### Data analysis

Data collected from OES and FEC induction was analyzed using Genstat 12th edition (VSN International, Hemel Hempstead, UK). Similarly, data collected on survival and growth of FEC and *in vitro* nodal cuttings exposed to gamma radiation was analyzed using Genstat 12th edition. All data was subjected to ANOVA and results were considered statistically significant at the 5% level.

## Results

### The effect of exogenous auxin and amino acids on production of organised embryogenic structures and friable embryogenic callus in the selected cassava genotypes

#### Production of organized embryogenic structures

**Figure 1:**
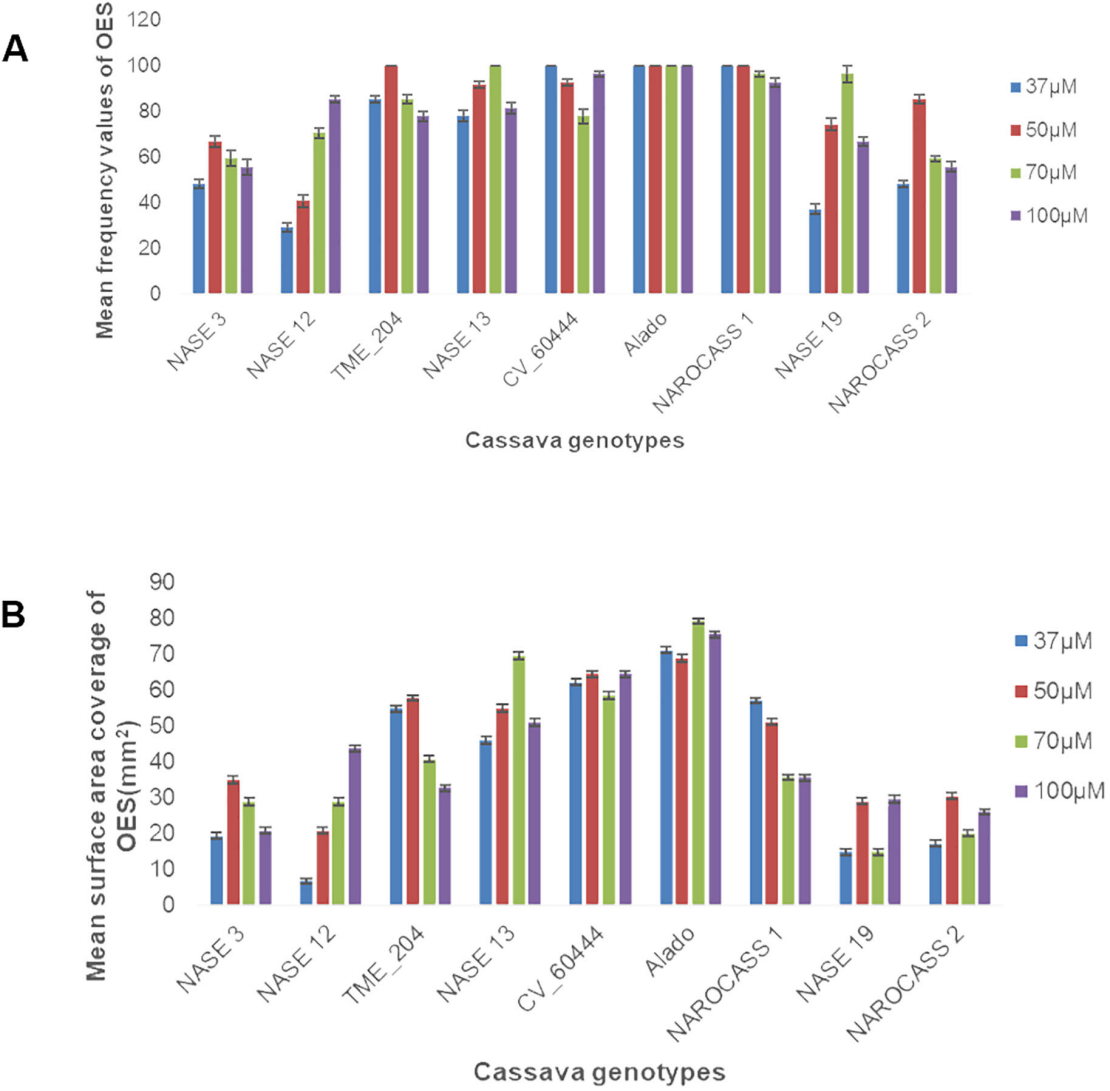
Frequency of production and surface area coverage of OES in UCGs **(A)** Effect of picloram concentration on frequency of OES production **(B)** Effect of picloram concentration on surface area coverage of OES produced at 28 days.

**Figure 2:**
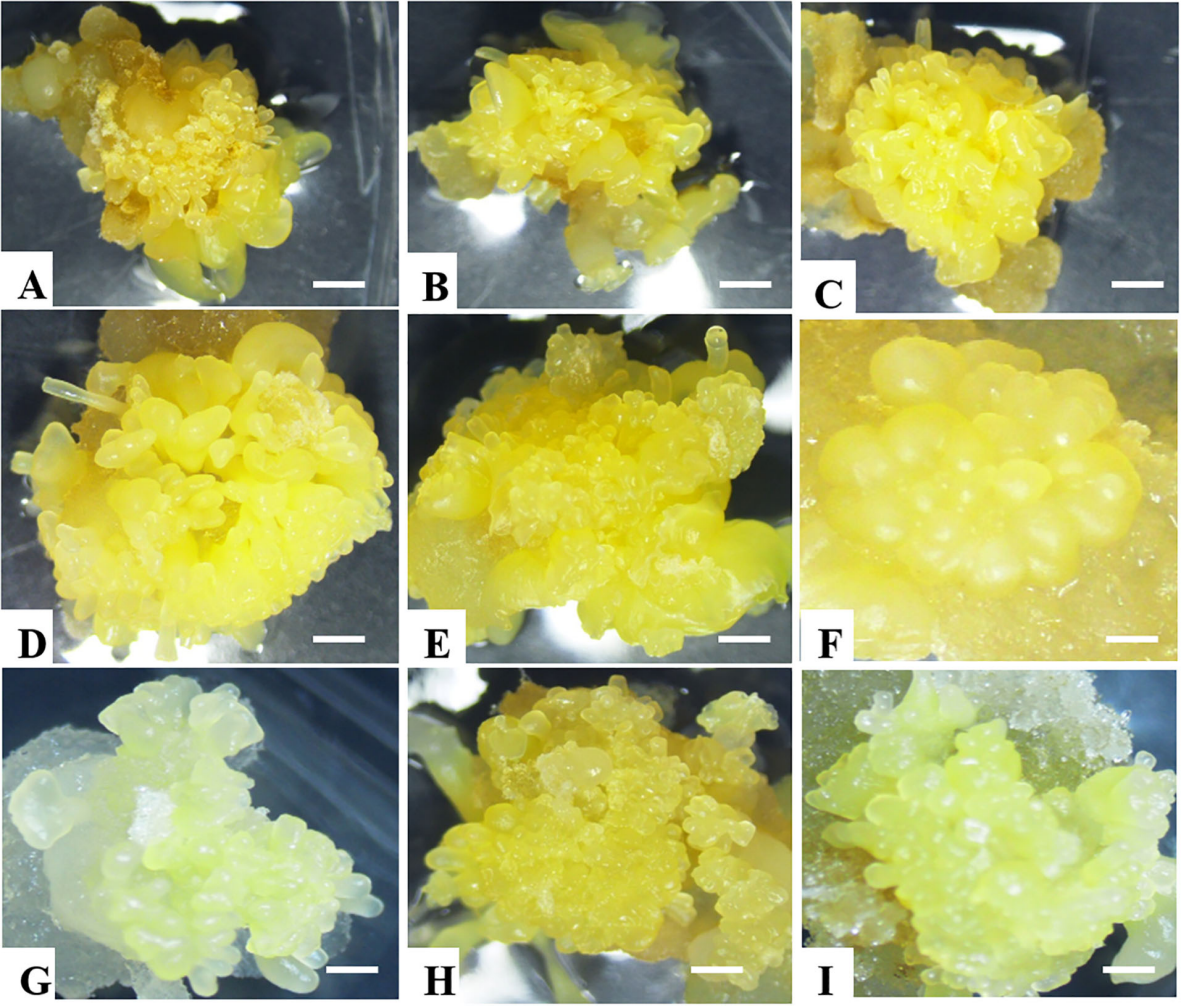
Somatic embryo production in all nine selected cassava genotypes. **(A)** NASE 12, **(B)** NASE 3, **(C)** NASE 13, **(D)** NASE 19, **(E)** Alado, **(F)** TME 204, **(G)** NAROCASS 1 **(H)** NAROCASS 2 and **(I)** CV-60444.

To facilitate the production of OES, leaf lobes were excised from each genotype and cultured on Murashige and Skoog (MS) medium supplemented with four different concentrations (37, 50, 70 or 100 µM) of picloram (an auxin analogue). All nine genotypes produced OES. However, the frequency and the surface area coverage of OES for each explant differed (**Figures 1A, B**, **2**). The frequency of production of OES was significant among genotypes (P = <.001) but not significant for the different picloram concentration (P = 0.051) and the interaction between genotypes and picloram concentration (P = 0.109). The genotype Alado had the highest percentage frequency across all the picloram concentrations investigated. The highest percentage frequency of OES production at 37 µM was observed in Alado, NAROCASS 1 and CV-60444 (100%), followed by TME 204 (85%), NASE 13 (78%), NASE 3 and NAROCASS 1 (48%), NASE 19 (37%) and least in NASE 12 (29%) (**Figure 1A**). In genotypes Alado, NAROCASS 1 and TME 204, the highest frequency was recorded at 50 µM (100%) followed by CV-60444 (93%), then NASE 13 (92%), NAROCASS 2 (85%), NASE 19 (74%), NASE 3 (67%) and the least in NASE 12 (41%) (**Figure 1A**).

#### Production of friable embryogenic callus

**Figure 3:**
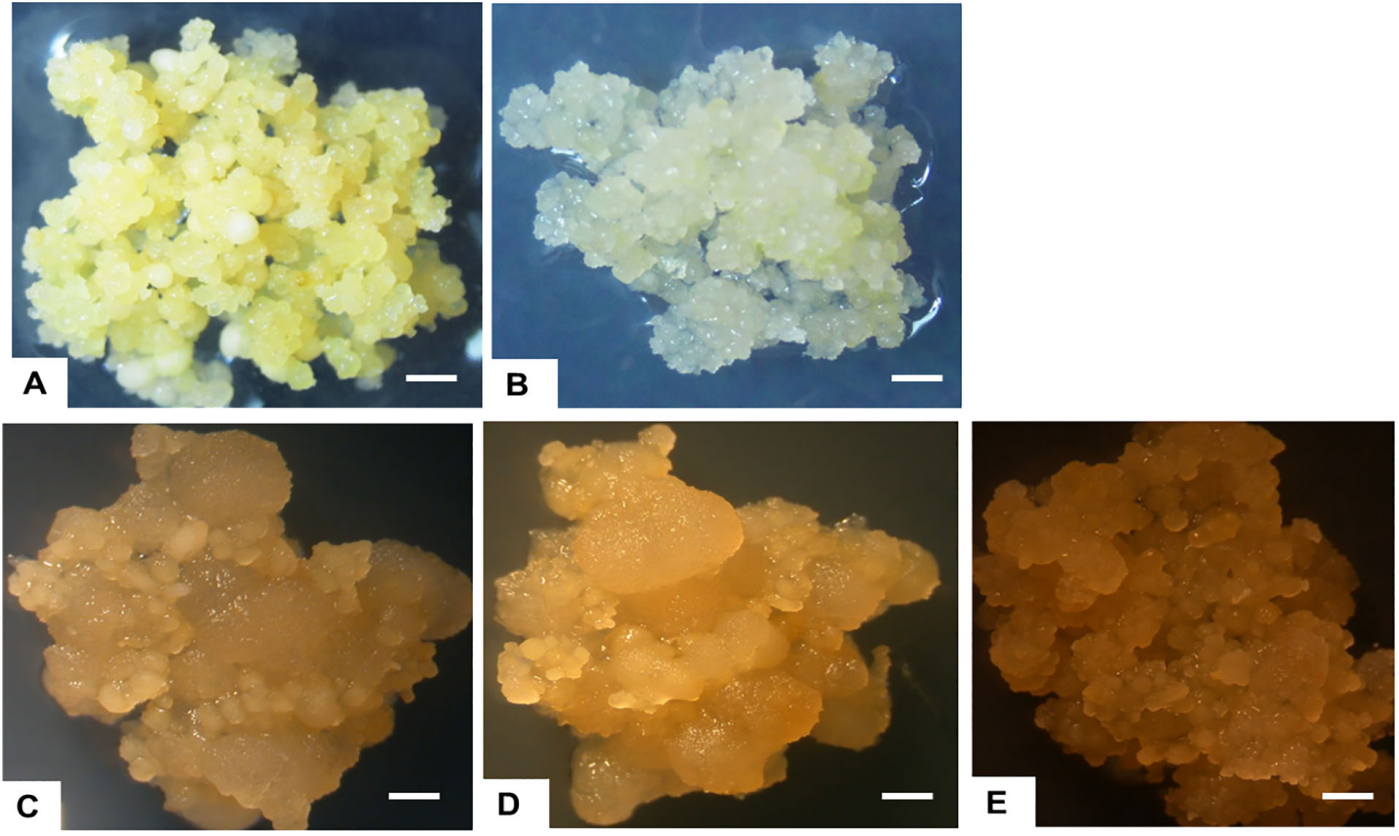
Formation of FEC tissues in UCGs and the model cultivar, CV-60444. **(A)** TME 204, **(B)** NASE 13, **(C)** Alado, **(D)** NASE 3 and **(E)** CV-60444.

To facilitate the production of FEC, 28-day-old OES was excised and crushed using a 1x1 mm^2^ wire mesh and cultured on Gresshoff and Doy (GD) basal media supplemented with two concentrations (250 or 500 µM) of the amino acid tyrosine at 28 ± 2°C. All five genotypes produced sufficient amounts of FEC as shown in **Table 1** **;** **Figure 3**. Production of FEC in the UCGs was dependent on the concentration of the amino acid, tyrosine, with the exception of the control cultivar CV-60444 (**Table 1**). The highest frequency of FEC production was observed in TME 204 (100%), followed by CV-60444 (60%), NASE 13 (31%), Alado (27%) and the least in NASE 3 (5%) (**Table 1** **;** **Figures 3A–E**). Production of FEC was successful in MS media ammended with 250 µM tyrosine for TME 204, NASE 3 and NASE 13 while in Alado, a higher concentration of tyrosine (500 µM) was required. FEC production in the control CV-60444 did not require the addition of tyrosine (**Table 1**).

**Table 1:** Production of FEC in different UCGs on tyrosine-amended medium.

Genotype	Media type	Frequency (%) of FEC produced
TME 204	GD2 50P, 250µM tyrosine	100.0
CV-60444	GD2 50P	60.0
NASE 13	GD2 50P, 250µM tyrosine	31.0
NASE 3	GD2 50P, 250µM tyrosine	05.3
Alado	GD2 50P, 500µM tyrosine	27.1

FEC frequencies were calculated by scoring the number of OES clusters that produced FEC/the total number of clusters per genotype.

### The effect of gamma radiation on mutation induction in FEC and nodal cuttings of UCGs

#### Effect of gamma irradiation on friable embryogenic callus

**Figure 4:**
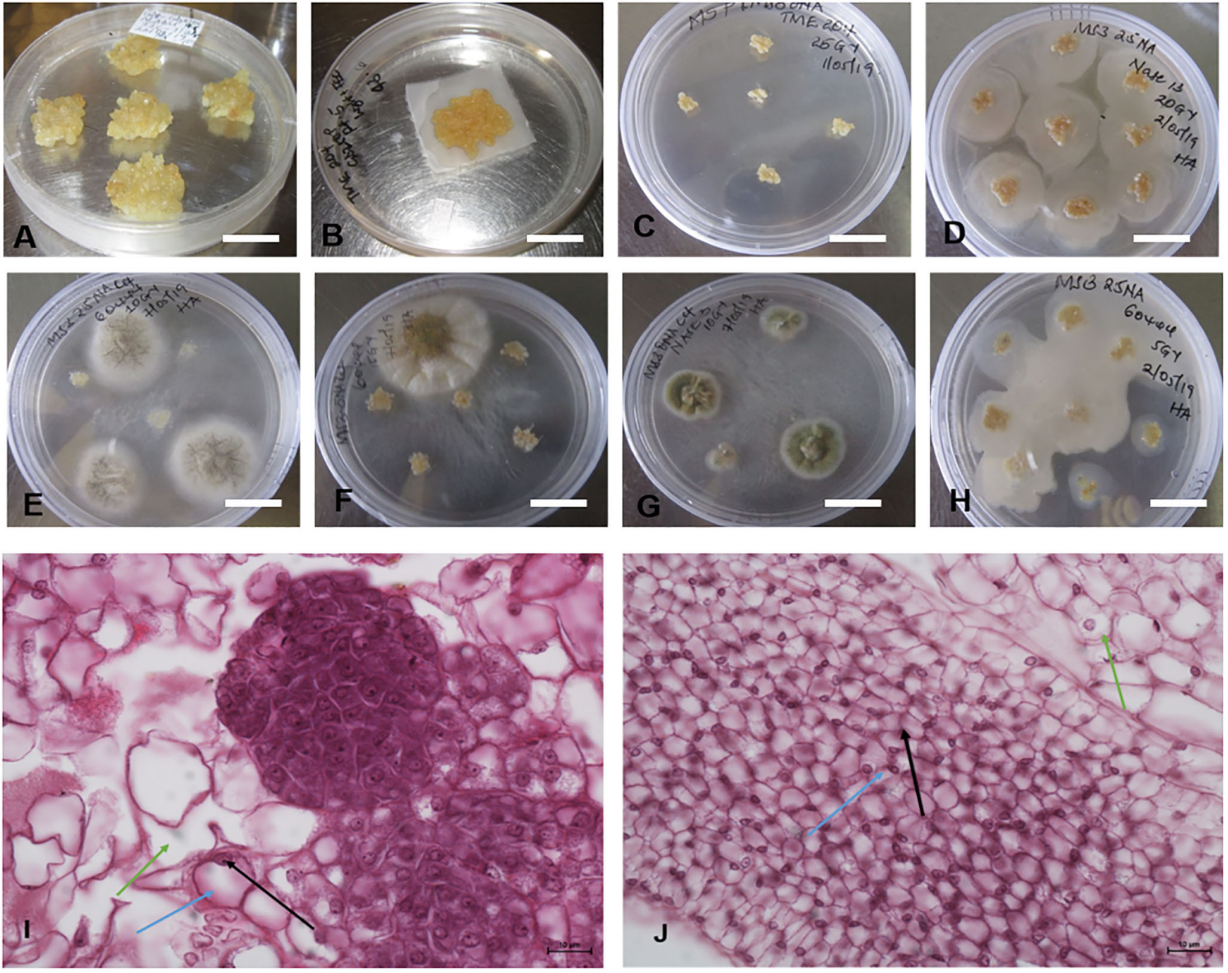
Different forms of contaminations observed in FECs exposed to different gamma doses. **(A)** Unirradiated FEC tissues **(B)** Sample of FEC tissue prepared for gamma radiation **(C)** Clean FEC of TME 204 irradiated at 25Gy at 28 days, **(D, F, H)**. Bacterial contamination observed in the different genotypes exposed to different radiation doses after 4 days and **(E, G)** Fungal contaminations observed in the different genotypes exposed to different radiation doses after 7 days. Differences were observed in the structure of cells in **(I)** unirradiated (control) and **(J)** irradiated friable embryogenic callus (FEC). Bar represents 5 mm **(A–H)** and 10μm **(I, J)**.

Sufficient amounts of FEC were required for replicability of the experiment (**Figure 4A**). The FEC was placed on sterile mesh to minimize its direct contact with media (**Figure 4B**). The effects of gamma irradiation on the growth of FEC from five different genotypes (TME 204, CV-60444, Alado, NASE 3 and NASE 13) were observed within the first four days. Regardless of the irradiation dose, cultures were overgrown by high bacteria and fungal-like contamination (**Figures 4C–H**). At 28 days of co-culture, irradiated cultures for all the genotypes were either lost or did not increase in size. For example, all the cultures (both controls and treatment) for genotype Alado were lost 28 days post-irradiation (dpi), while for CV-60444, only FEC cultures irradiated at 20 and 25 Gy were contaminated. (**Figures 4E, F, H**). In genotypes, NASE 3 and NASE 13, cultures irradiated at 5 Gy and the control survived on media supplemented with either 27 µM or 13 µM of NAA at 28 dpi. For TME 204, only cultures irradiated at 25 Gy survived on media supplemented with either 27 µM ml/L or 13 µM at 28 dpi. Unfortunately, all surviving cultures succumbed to contamination at 35 dpi in culture.

Histological analysis of FEC post-gamma irradiation revealed differences in the structure of the cells in irradiated tissues and the control (**Figures 4I, J**). The pink color is a result of eosin that stains the cytoplasm while the dark blue stains are attributed to hematoxylin which stains acidic structures like the nucleolus (indicated by the black arrow), the blue arrow shows the nucleus and the green arrow, the cytoplasm. The structure of the cells in the control FEC tissues was well-defined and large and the nucleus was at the periphery of the cell (**Figure 4I**). In contrast, in the irradiated FEC tissues, the cells appeared smaller and the nuclear material was not prominent (**Figure 4J**). There appear to be dead embryogenic cells with abnormal pigmentation (**Figure 4J**).

#### Effect of gamma radiation on shoot survival of *in vitro* nodal cuttings

**Figure 5:**
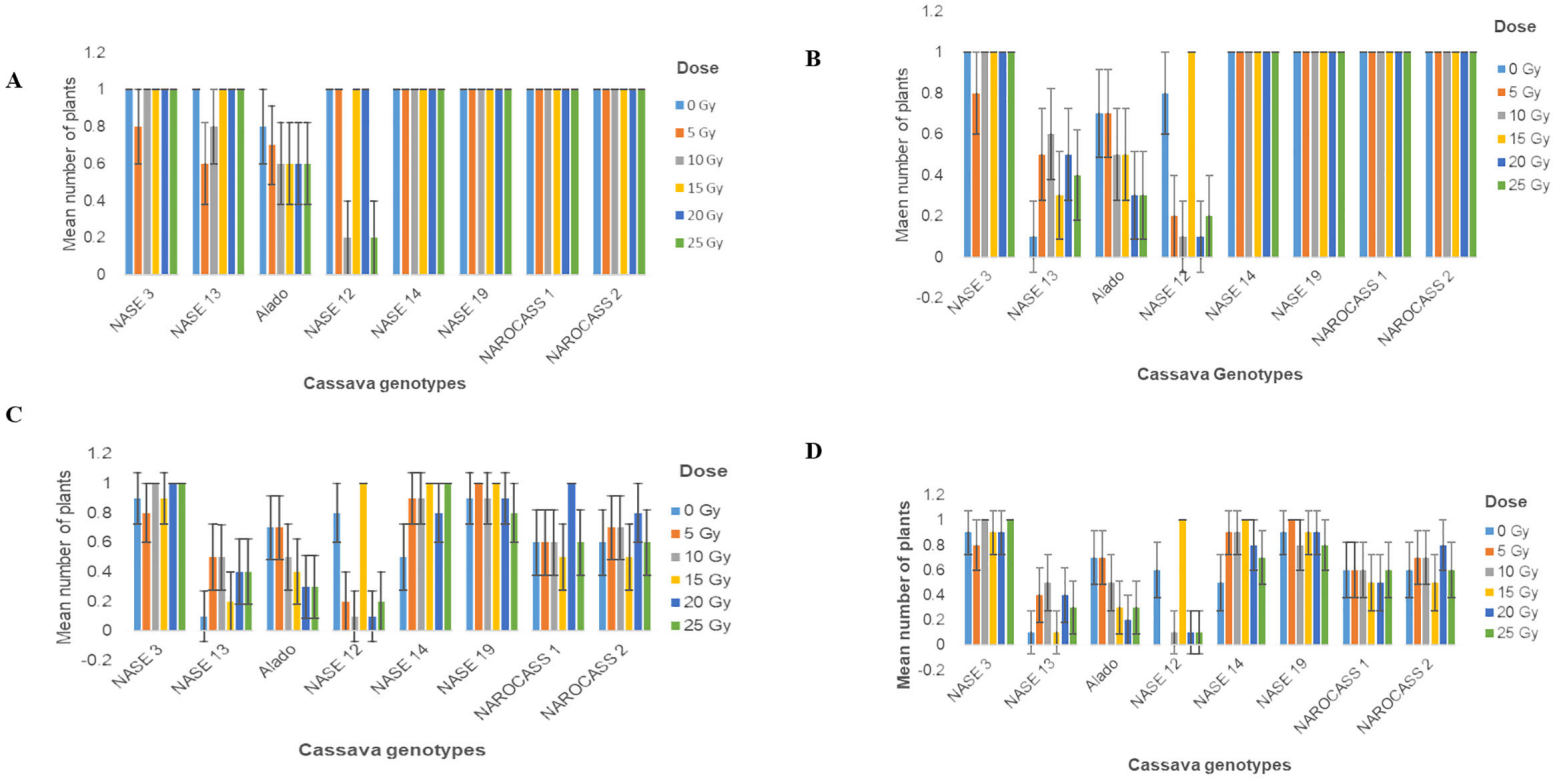
Number of surviving plants in eight UCGs exposed to different doses of gamma radiation at **(A)** 4; **(B)** 7; **(C)**14 and **(D)** 21 days in culture post irradiation.

The effect of gamma radiation on *in vitro* nodal cuttings was observed in all for the different genotypes for the number of plants that survived. There was a decrease in the number of irradiated cultures over time (**Figures 5A–D**). General loss of plants was observed in NASE 13, Alado and NASE 12 at 14 and 21 dpi (**Figures 5C, D**). At 28 days of culture, NASE 3, NASE 14, NASE 19, NAROCASS 1 and NAROCASS 2 had at least 5 or more plants surviving (**Table 2**).

**Table 2:** Number of plants of each genotype that survived after exposure to different gamma radiation doses at 28 days of culture.

Genotypes	Gamma radiation doses/number of surviving plants*					
	0 Gy	5 Gy	10 Gy	15 Gy	20 Gy	25 Gy
NASE 3	08	08	10	07	09	10
NASE 13	01	04	05	01	03	02
Alado	07	07	05	03	02	01
NASE 12	06	00	01	10	01	01
NASE 14	05	09	09	10	08	06
NASE 19	09	10	08	09	09	08
NAROCASS 1	06	06	06	05	05	06
NAROCASS 2	05	06	07	05	08	06

*Total number of plants that survived after exposure of in vitro nodal cuttings to different gamma radiation doses.

**Figure 6:**
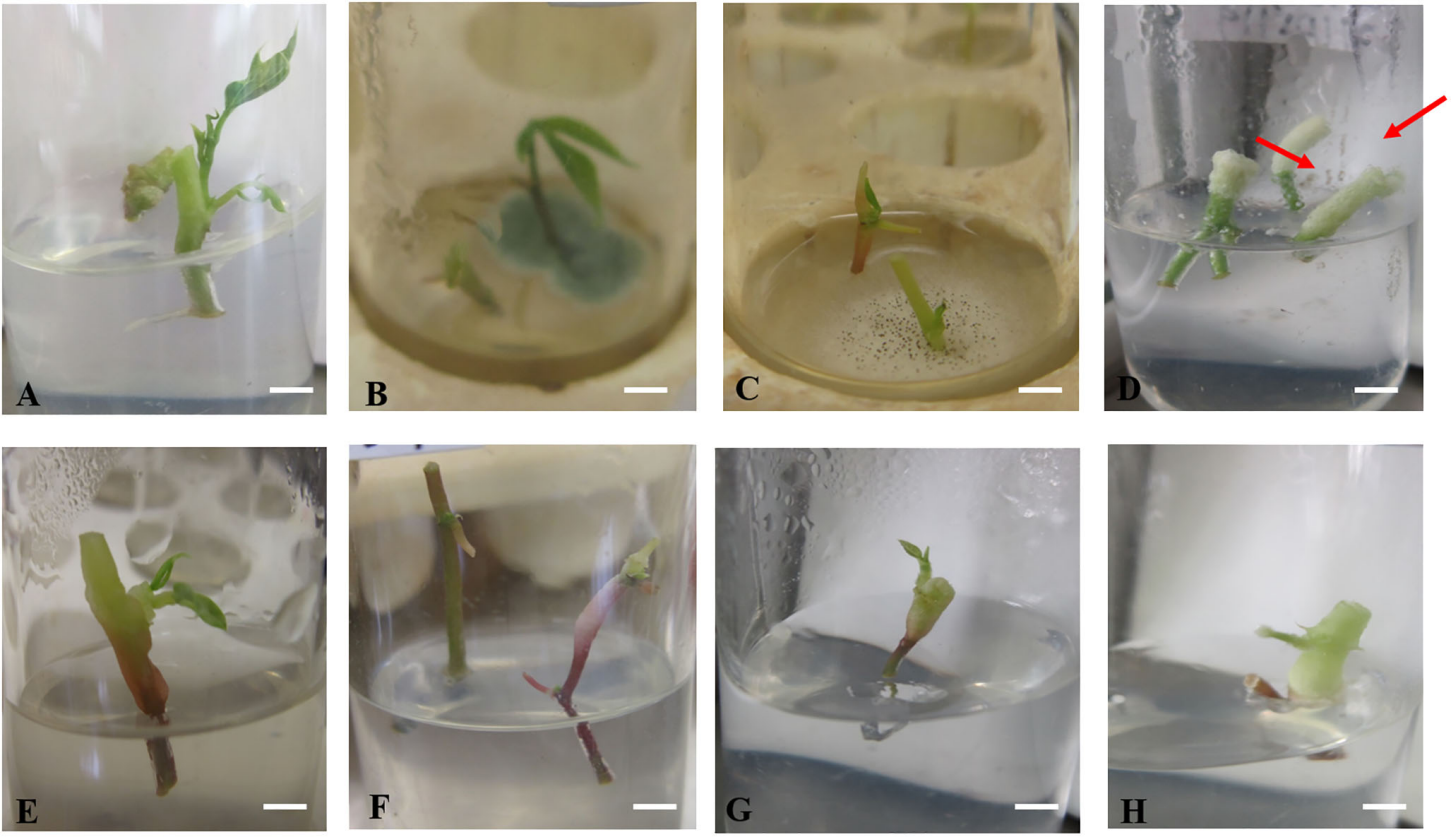
Response of irradiated *in vitro* nodal cuttings of selected Ugandan genotypes 4 days after culture on MS medium. **(A)** Clean nodal cuttings of Alado, **(B)** blue fungal contamination, **(C)** fungal and bacterial growth around nodal cuttings **(D)** white callus tissue on the top part of the nodal cutting (arrowed), **(E)** loss of purple color at the upper portion of nodal cutting, **(F)** Bulging observed on the nodal cuttings, **(G)** bulging of the upper part of the nodal cutting and **(H)** discoloration and thickening of the top of nodal cutting.

For *in vitro* nodal cuttings, some of the cultures were contaminated after four days, while others grew a bulge (**Figures 6E–H**), or white callus at the point of excision (**Figure 6D**), indicative of the effect of gamma irradiation on cassava *in vitro* nodal cuttings. For some genotypes (NASE 14, NASE 19, NAROCASS 1 and NAROCASS 2) all 10 plants survived after 7 dpi in culture for the different radiation doses (**Figures 5A, B**). At 28 days of culture, the highest number of surviving plants in the unirradiated control plants were observed in NASE 19 (9), followed by NASE 3 (8) and the least in NASE 13 (1) (**Table 2**). At 5 Gy, the highest number of surviving plants was in NASE 19 (10) and the least in NASE 12. At 15 Gy, the highest number of surviving plants was observed in NASE 12 (10) and NASE 14 (10), followed by NASE 19 (9), then NASE 3 (7), NAROCASS 1 (5), NAROCASS 2 (5), Alado (3) and least in NASE 13 (1) (**Table 2**).

### Effect of gamma radiation on root growth of *in vitro* nodal cuttings

**Figure 7:**
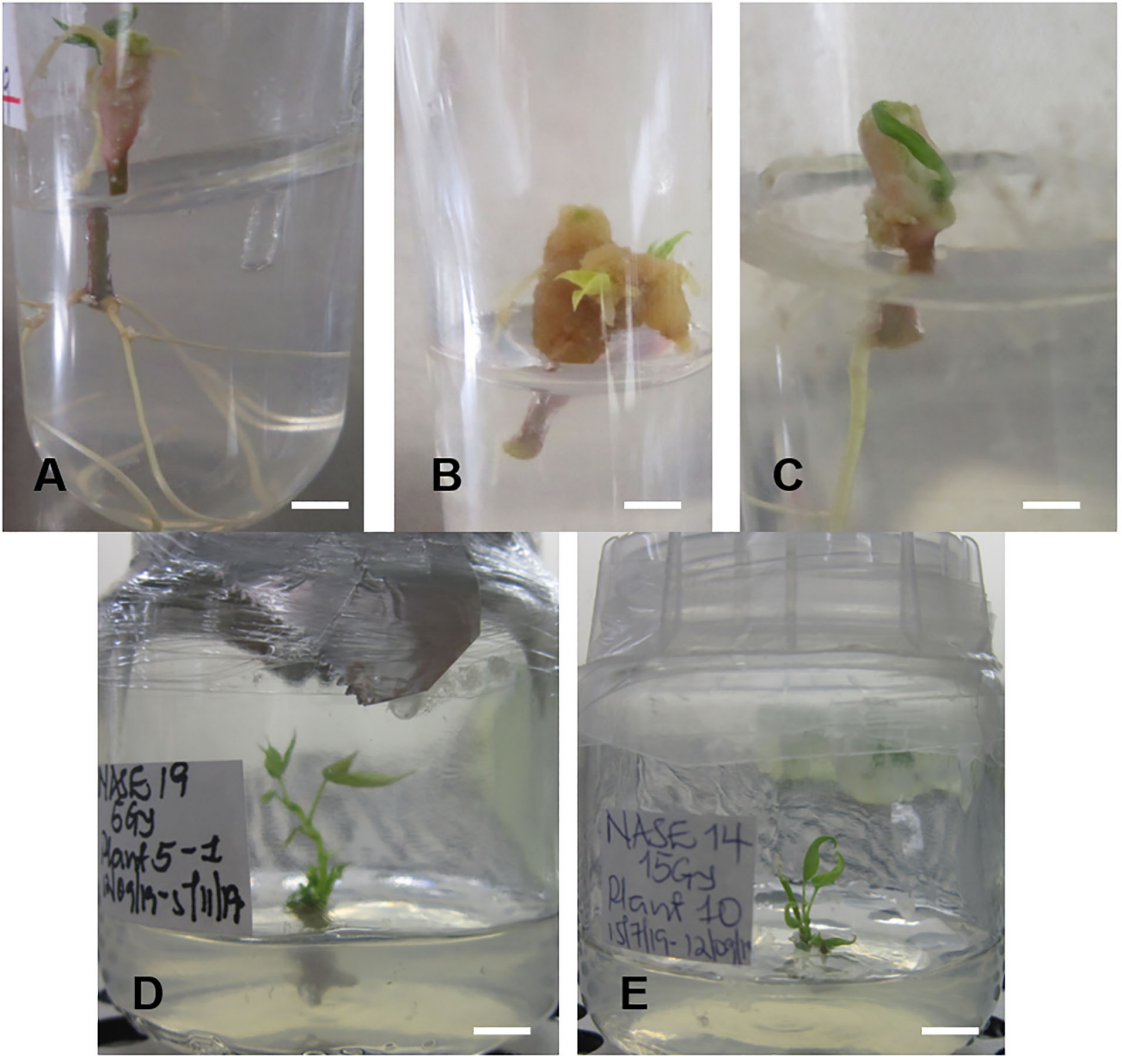
Root formation of irradiated *in vitro* nodal cuttings (ivNC) of Alado after exposure to three different doses and regenerated (ivNC) of NASE 19 and NASE 14 six months post-irradiation. **(A)** 5 Gy, **(B)** 10 Gy, **(C)** 15 Gy, **(D)** Regenerated ivNC of NASE 19 with roots at 5 Gy and **(E)** Regenerated ivNC of NASE 14 with no roots at 15 Gy.

The irradiated *in vitro* nodal cuttings did not increase in height, instead produced roots as shown in **Figures 6** and **7**. This suggests that gamma irradiation negatively impacted plant growth responses such as shoot growth, acting similarly to auxin efflux inhibitors in the shoots while root development dependent on unidirectional auxin movement was less affected (Tanaka et al., 2006). All the genotypes tested produced roots between 14 – 28 days of culture (**Table 3**). The number of roots varied from genotype to genotype at the different radiation doses (**Table 3**). At 0 Gy (control), the highest number of roots were produced by Alado (18) followed by NASE 12 (12), then NASE 14 (9), NASE 3 (8), NAROCASS 1 (7), NASE 19 (6), NAROCASS 2 (1) while NASE 13 did not produce roots at 28 days in culture (**Table 3**). At 5 Gy, the highest number of roots produced were observed in NASE 14 (9), followed by NASE 3 (8), NASE 19 (8), Alado (5), NASE 13 (3), NAROCASS 1 (1) and no roots were produced by NASE 12 and NAROCASS 2. At 10 Gy, the highest number of roots were observed in Alado (11) followed by NASE 3 (9), NASE 14 (7), NASE 19 (4), NASE 13 (1), NAROCASS 1 (1) and none in NASE 12 and NAROCASS 2 (**Table 3**). At 15 Gy, only three genotypes produced roots. The highest number of roots were produced by NASE 14 (12) followed by NASE 12 (9) then NASE 19 (6). Similarly, at 20 Gy only three genotypes, NASE 19 (4), NASE 3 (3) and Alado (1) produced roots (**Table 3**). NAROCASS 2 (2) was the only genotype that produced roots at 25 Gy after 28 days of culture. Subsequently, surviving roots of NASE 14 (15 Gy) and NASE 19 (5 Gy) were sub-cultured onto fresh MS medium to promote shoot and root growth as shown in **Figures 7D, E**.

**Table 3:** Total number of roots produced by each genotype after exposure to five different radiation doses after 28 days in culture.

Genotypes	Gamma Radiation doses/Number of Roots produced*					
	0 Gy	5 Gy	10 Gy	15 Gy	20 Gy	25 Gy
NASE 3	08	08	09	00	03	00
NASE 13	00	03	01	00	00	00
Alado	18	05	11	00	01	00
NASE 12	12	00	00	09	00	00
NASE 14	09	09	07	12	00	00
NASE 19	06	08	04	06	04	02
NAROCASS 1	07	01	01	00	00	00
NAROCASS 2	01	00	00	00	00	00

*Roots produced in each genotype after exposure of in vitro nodal cuttings to gamma radiations doses.

### Determination of LD_50_ for *in vitro* nodal cuttings of UCGs

**Figure 8:**
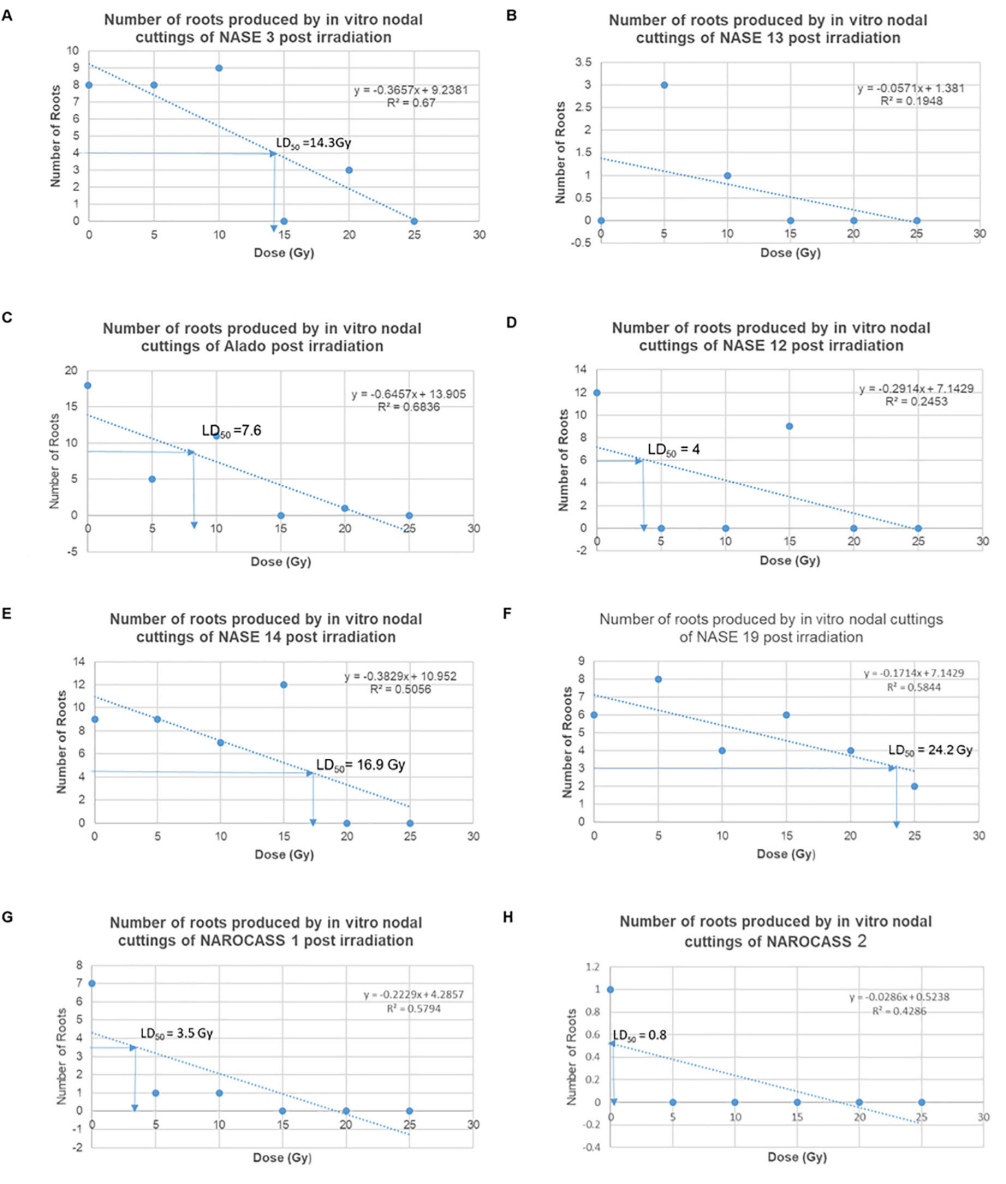
Graphs showing the trend of the number of roots produced per genotype with different doses and their calculated LD_50._ **(A)** NASE 3, **(B)** NASE 13, **(C)** Alado, **(D)** NASE 12, **(E)** NASE 14, **(F)** NASE 19, **(G)** NAROCASS 1 and **(H)** NAROCASS 2.

The average number of roots produced by nodal cuttings of UCGs in relation to the different radiation doses was used to calculate the LD_50_ values using the linear regression equation, which are graphically determined. Based on this data, the LD_50_ estimated for NASE 3, Alado, NASE 12, NASE 14, NASE 19, NAROCASS 1 and NAROCASS 2 were 14, 5, 5, 14, 22, 4, and 8 respectively in that order (**Figure 8**). No LD_50_ was for NASE 13 because the control did not produce roots. The highest correlation value was observed in genotype NASE 3 (0.67), followed by Alado (0.65), then NAROCASS 1 (0.58), NAROCASS 2 (0.39), NASE 14 (0.36), NASE 19 (0.29) and least in NASE 12 (0.29) (**Figure 8**).

### Gamma radiation has an effect on regeneration of *in vitro* nodal cuttings of cassava

**Figure 9:**
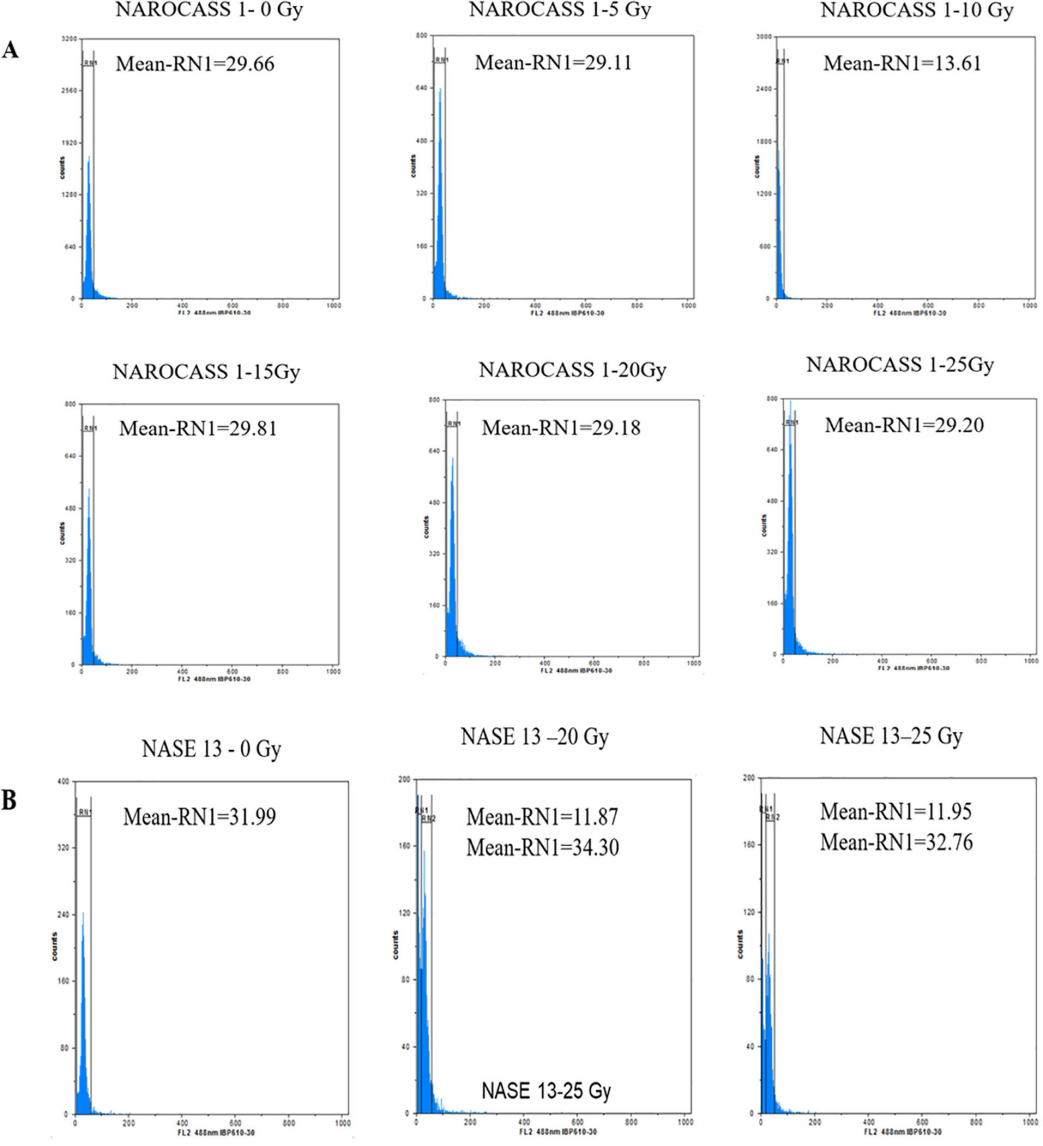
Flow cytometry analysis showing relative fluorescence intensity of *in vitro* nodal cuttings of cassava genotypes **(A)** NAROCASS 1 and **(B)** NASE 13 exposed to different doses of gamma radiation. Biological replicates, n = 6. Each histogram shows the nuclear DNA level for each leaf sampled.

To ascertain the impact of gamma irradiation on nodal cuttings that survived, flow cytometry was used to determine the ploidy status of nodal cuttings and changes that may have occurred in the genome of the plant after treatment with gamma radiation (**Figures 9A, B**). All eight genotypes produced DNA histograms that indicated that they still had a diploid state. In comparison to the controls, the irradiated nodal cuttings from Alado, NASE 12, NASE 19, and NASE 14 had a shift of the peak mean values to the left for all the irradiation doses tested (**Table 4**). For NASE 3 at 5 Gy and 10 Gy, the peak mean values obtained presented a shift to the right, probably indicating cell activity. NAROCASS 1 indicated a loss of half the genomes at 10 Gy in comparison to the control as shown in **Figure 9A**. The production of double peaks was observed in NASE 13 and NAROCASS 2 at 20 Gy and 25 Gy respectively, in comparison to the control as shown in the **Figure 9B** (representing DNA histograms for NASE 13).

**Table 4:** Data showing the mean peak values of the different genotypes when exposed to different radiation concentrations in relation to the control.

Genotypes	Radiation doses/Calculated Mean Peak Values*					
	0 Gy	5 Gy	10 Gy	15 Gy	20 Gy	25 Gy
Alado	30.82	26.18	28.50	29.82	30.48	28.66
NAROCASS 1	29.65	29.11	13.61	29.81	29.18	29.20
NASE 13	31.99	25.92	31.63	29.96	**11.87**	**11.95**
NASE 14	32.09	31.64	31.42	29.99	29.12	29.61
NASE 19	32.32	31.66	31.58	29.81	00.00	31.87
NAROCASS 2	28.40	29.26	28.64	30.73	**11.41**	**12.85**
NASE 3	31.69	34.36	33.72	30.66	30.59	29.81
NASE 12	28.51	00.00	27.14	28.53	00.00	26.91

*Mean Peak Values are calculated from the Sysmex Partec GmbH flow cytometry program.

The bold values indicate that half the genome of the plant was lost when compared to the control at 0 Gy.

## Discussion

Induced mutations have been used as an alternative strategy to introduce desirable traits into several crop species (Forster and Shu, 2012). The exposure of multicellular plant parts to mutagens results in the generation of chimeric plants which require further cycles of propagation to obtain solid mutants. The use of *in vitro* plants or embryogenic tissues such as FEC generated via somatic embryogenesis as target material for mutation induction in cassava can reduce the risk of generating chimeric plants.

In this study, four concentrations of picloram (4-Amino-3,5,6-trichloropicolinic acid), a synthetic auxin that has been used for somatic embryogenesis in several African cassava genotypes was used (Danso et al., 2010; Apio et al., 2015; Mongomake et al., 2015; Nyaboga et al., 2015; Elegba et al., 2021). In MS medium supplemented with picloram, the production of organized embryogenic structures (OES) was possible in UCGs albeit variation in the amount and frequency of primary somatic embryo production (Berleth and Sachs, 2001; Fehér, 2015). In cassava, the supplementation of MS medium with picloram has been extensively used for the induction of OES in several African cassava genotypes (Taylor et al., 2012; Nyaboga et al., 2013; Apio et al., 2015; Mongomake et al., 2015; Danso and Elegba, 2017). In plants, *de-novo* biosynthesis of auxins (Indole 3-Acetic acid, IAA) is essential for embryogenesis, particularly at the beginning of the somatic embryogenesis (SE) induction process (Uc-Chuc et al., 2020). The SE process in plants is genotype-dependent and triggered in the presence of plant growth regulators (Desai et al., 2022; Kępczyńska and Kępczyński, 2023). Furthermore, SE is a stress-related process that results in changes in the expression of responsive genes through locus-specific regulation of DNA methylation in plants (Fehér, 2015). The transition of somatic embryos to FEC has been associated with the upregulation or downregulation of plant hormone signaling pathways (Ma et al., 2015). Besides FEC induction, the color as well as the time taken to produce FEC is influenced by the genotype. In the Ugandan genotypes research has shown that substantial amounts of FEC were produced only after 35 days in comparison to 21- 28 days in the control genotype, 60444 (Apio et al., 2015). Another factor essential for SE/FEC induction is the presence of nitrogen and phosphate in the medium that is required for proper auxin signaling, composition of phospholipids and DNA structure in cells (Lambers, 2022). MS medium has a nitrogen-to-phosphate (N: P) ratio of 40:1 and has been shown to support the routine production of OES in cassava (Taylor et al., 2001; Bull et al., 2009; Apio et al., 2015; Elegba et al., 2021). According to Pasternak and Steinmacher (2024) the optimal ratio of nitrogen to phosphate (N: P) for most plant species is 5 - 6: 1 for shoot induction and 10-12: 1 for plant growth.

Our results confirmed the genotype-dependent response of Ugandan genotypes to OES production with the genotype Alado producing the highest OES for all four picloram concentrations (37, 50, 70, 100 µM) used. Similarly, the optimum picloram concentration for OES production was higher (70 µM) in NASE 12 and NASE 13) in contrast to the other six genotypes (NASE 3, TME 204, CV-60444, NASE 19, NAROCASS 1 and NAROCASS 2) where it was at 50 µM, in agreement with earlier work on UCGs (Apio et al., 2015). The response of the genotypes to the different concentrations of picloram suggests that auxins are key in cellular reprogramming of plant tissues, genetically, metabolically and physiologically, resulting in embryogenic competence (Fehér, 2015, 2019). Also, the ability of the different genotypes to produce somatic embryos suggests that the synthetic auxin picloram is an effective tool for induction of endogenous auxin synthesis and canalisation sufficiently mimicking IAA, the naturally-occurring auxin that plays an essential role in all aspects of plant life including somatic embryogenesis (Armengot et al., 2016; Uc-Chuc et al., 2020).

The transfer of OES tissues to GD medium supplemented with picloram and tyrosine resulted in FEC production in the UCGs. The transition of somatic embryos to FEC is highly dependent on dedifferentiation in which transcriptional and translational profiles are altered to allow cells to enter a new developmental pathway (Fehér, 2015). Cell differentiation and dedifferentiation are regulated by genetic and epigenetic mechanisms resulting in callus induction affecting gene expression via chromatin modification including DNA methylation and histone modification (Ma et al., 2015). FEC induction in cassava, particularly African varieties has been strongly influenced by genotype with some genotypes recalcitrant to the process (Chetty et al., 2013; Nyaboga et al., 2013; Apio et al., 2015; Elegba et al., 2021). The production of FEC in the control genotype 60444 did not require tyrosine in contrast to FEC production in TME 204, NASE 13 and NASE 3, which was possible only in GD medium supplemented with tyrosine at a concentration of 250 µM. The supplementation of GD medium with tyrosine has been reported to improve the conversion of OES to FEC in some African cassava genotypes (Nyaboga et al., 2013; Apio et al., 2015; Nyaboga et al., 2015). For the genotype Alado, tyrosine at a higher concentration (500 µM) was required to enhance the conversion of OES to FEC as earlier reported (Apio et al., 2015). However, the positive effect of tyrosine on FEC induction is genotype-dependent and not all genotypes respond positively (Nyaboga et al., 2013). Amino acids are precursors of several plant hormones and their addition to the medium replaces ammonium ions, thereby increasing the levels of nitrogen which is required for the development of somatic embryos (George et al., 2008). In particular, the amino acid tyrosine plays a key role in regulating tyrosine phosphorylation and has been implicated in many signaling pathways and plant physiological processes (Shankar et al., 2015).

The effects of the exposure of FEC to varying doses of gamma irradiation were noted after four days irrespective of radiation dose. Gamma radiation is preferred for mutation induction because of its ease of use and shorter wavelength, thus, high power of penetration (Moussa, 2006; Marcu et al., 2013). Our results indicate that exposure of embryogenic cells (FEC) to gamma radiations results in the death of cells. Cells contain around 90% water and exposure of cells to gamma radiation induces chain reactions that produce secondary free radicals (Marcu et al., 2013; Golz and Bradshaw, 2019). Among the processes that occur include base excision, DNA replication, and nucleotide excision repair resulting in DNA damage or repair (Ma et al., 2015). Gamma irradiation-induced oxidative stress results in the overproduction of reactive oxygen species (ROS) such as superoxide radicals, hydroxyl radicals, and hydrogen peroxides, which results in the modification of crucial components in plant cells, disrupting vital biochemical and physiological processes essential for the survival of the plant cells and tissues (Esnault et al., 2010; Marcu et al., 2013). This likely resulted in the death of embryogenic tissues and *in vitro* nodal cuttings post-irradiation.

In the case of the *in vitro* nodal cuttings (ivNCs), exposure to gamma irradiation resulted in swelling at points along the explant without an increase in height except for the controls. In some instances, the point of contact of the irradiated explant with the medium was colonized by bacteria, fungus or both. These responses could be attributed to the type of explant. *In vitro* nodal cuttings are young tender tissues compared to cassava stakes that are hard and robust and have been the main plant tissues routinely used as target tissues for mutation induction (Asare and Safo-Kantanka, 1995; Khumaida et al., 2015; Danso and Elegba, 2017; Baguma et al., 2021). Gamma radiation produces single-strand breaks (SSBs) in the DNA of cells and if not repaired leads to failure of DNA replication and, ultimately cell death (Chatterjee and Walker, 2017). A significant reduction in the number of leaves and nodes, weight and length of *in vitro* plants of cassava after exposure to different doses of gamma radiation has been reported (Ndofunsu et al., 2015). The number of roots produced per genotype was influenced by gamma irradiation doses with higher doses impeding root formation in most genotypes compared to their corresponding non-irradiated controls, which produced roots. In Arabidopsis, exposure of roots to unilateral ultraviolet (UV) radiation (UV-B) reduces auxin levels and leads to an asymmetric distribution of auxin in root tips (Yokawa et al., 2016; Wan et al., 2018).

The lethal dose range (LD_50_) was determined using the number of roots, given that there was no increment in plant height at 30 days post-irradiation. The calculated LD_50_ values obtained varied from genotype to genotype which is in harmony with results obtained from physic nut (Songsri et al., 2011). The data indicates that low dosages (5 – 15 Gy) allowed for the production of roots compared to high dosages (20 - 25 Gy) across genotypes. This result is in agreement with work in several plant species in which low-dose irradiation had a positive effect on root growth and length (Charbaji and Nabulsi, 1999; Jan et al., 2011).

To determine the DNA content and ploidy status of *in vitro* plants of UCGs after exposure to gamma irradiation, flow cytometry technique was used. The histogram peaks obtained were a reflection of the number of molecules that were bound to the probe which translates to the number of molecules of DNA present in a sample (Ochatt, 2008). In flow cytometry, knowledge of the genome size is important for the characterization of induced variation and establish ploidy status (Natarajan et al., 2023). Majority of the genotypes had DNA histograms with one peak similar to the control and corresponding to the nuclei in G1 phase of mitosis. This indicates that gamma irradiation did not cause changes to the DNA content in these genotypes. However, in the genotype NAROCASS 1 the mean values obtained from plants exposed to different doses of gamma irradiation (5, 20, 25 Gy), revealed a shift to the left compared to the control (0 Gy), indicating loss of genetic information or deletions in the genome. In the same genotype, half of the genome was lost at 10 Gy based on the low mean value, resulting in loss of genetic information as well as the ability of the explant to survive because cell division ceased (Ochatt, 2008; Natarajan et al., 2023). At 15 Gy, the mean values shifted to the right indicating cell activity related to biochemical and physiological activities. The effect of different doses of gamma irradiation on mutation induction in cassava is genotype-dependent as recorded in this study. Thus, it is important to characterize plant tissues via flow cytometry or sequencing post-irradiation to identify putative mutants or mutations.

In NASE 13, at gamma doses of 20 and 25 Gy) two mean peak values were obtained. The peaks with values close to the control were likely in the G0/G1 phase. However, the smaller peaks with low mean values were indicative of cell death as a result of gamma irradiation (Ochatt, 2008). The exposure of deficient HCT116 colon cancer cell lines to gamma irradiations at a dose of 5 Gy produced two peaks, the first peak was identified as having apoptotic cells, whereas the second peak was of cells at the G1 phase in the irradiated cell lines (Halacli et al., 2013). Doses lower than 5 Gy have been documented to be more reliable using acute and chronic gamma irradiation (Caplin and Willey, 2018). However, the lower doses were not consistent and reliable with the Cobalt-60 machine that was used for the experiment. In the case of Wild Musa species from India, peaks were observed that were indicative of the G1 and doubling of the chromosome showing the ploidy status of the species (Natarajan et al., 2023).

## Conclusions

Gamma irradiation at doses of 5 to 25 Gy affect the growth of FEC and *in vitro* nodal cuttings in different cassava genotypes. The biochemical and physiological changes in plant tissues due to gamma radiation resulted in variation that was either useful or detrimental to plant growth and development. The death of tissues over time and the decrease in root formation of *in vitro* nodal cuttings highlight the potency of gamma irradiation for the induction of variation in cassava. The optimization of the radiation doses for *in vitro* tissues would allow for the recovery of plants for further screening in the field. Although gamma irradiation affected plant growth (shoots and roots) and survival, the ploidy level was maintained in surviving tissues except in NAROCASS 1 at 10 Gy. The information reported in this work documents for the first time the effect of gamma irradiation on the survival and growth of *in vitro* tissues (nodal cuttings and FECs) in cassava in comparison to the use of cassava stakes which have been routinely used as target materials for mutation induction in the crop. This work will serve as a guide for further studies aiming to exploit *in vitro* tissues for mutation induction in cassava.

## Data Availability

The original contributions presented in the study are included in the article/supplementary material. Further inquiries can be directed to the corresponding authors.
